# Keeping your senescent cells under control

**DOI:** 10.18632/aging.100046

**Published:** 2009-05-06

**Authors:** Lars Zender, K. Lenhard Rudolph

**Affiliations:** ^1^Helmholtz Centre for Infection Research, Braunschweig, Germany and Department of Gastroenterology, Hepatology & Endocrinology, Hannover Medical School, Germany; ^2^Institute of Molecular Medicine and Max-Planck-Research Group on Stem Cell Aging, University of Ulm, Germany

**Keywords:** Senescence associated secretory phenotype, SASP, stress signaling, Il-6, telomeres, oncogene induced senescence

Cellular senescence is a stable form of cell-cycle arrest which is thought to limit the proliferative potential of premalignant cells [1]. The senescence phenotype was initially described by Hayflick and Moorhead in 1961 on human fibroblasts undergoing replicative exhaustion in culture [2]. It has been shown that senescence can be triggered in different cell types in response to diverse forms of cellular damage or stress (for review see [1]). Importantly, while senescence was denounced as a tissue culture phenomenon for many years, recent *in vivo* studies demonstrated that cellular senescence represents a potent failsafe mechanism against  tumorigenesis and contributes to the cytotoxicity of certain anticancer agents (see for example [3-7]). Interestingly, senescent cells have also been observed in certain aged or damaged tissues and there is growing evidence that senescence checkpoints can affect the regenerative reserve of tissues and organismal aging [8-11]. However, senescence may also have positive effects on organ maintenance by limiting pathological responses to acute forms of injury such as fibrotic scarring in response to chemical induced liver injury [12].

Over the past years it was also shown that senescent cells can communicate with their environment by secreting a myriad of cytokines and growth factors. Interestingly, this "senescence associated secretory phenotype (SASP)" seems to be a double edged sword regarding tumor initiation and maintenance:

i) On the one hand, it has been shown that the SASP can have pro-tumorigenic effects. In an experimental system it was shown that senescent mesenchymal cells can enhance the tumorigenicity of surrounding breast cancer cells [13].

ii) Similarly, it is possible that the SASP enhances selection of transformed cell clones in aged organ systems. It has been shown that loss of proliferative competition of non-transformed cells can accelerate leukemogenesis [14]. It remains to be seen whether aberrant secretion of cytokines and growth factors by the SASP can accelerated this process in aged and chronically damage organ systems.

iii) In contrast to its pro-tumorigenic aspect, the SASP could also have anti-tumor effects.  A recent study showed that in a mosaic liver cancer mouse model the activation of p53 induced senescence, an upregulation of inflammatory cytokines, and activation of innate immune responses leading to tumour cell clearance [15].

iv) In further support that the SASP could have anti-tumor activities, a series of recent papers showed that components of the SASP can stabilize the senescence cell cycle arrest via an autoregulatory feedback loop [16,17] or induces apoptosis of tumor cells [18].

In addition to its effects on tumorigenesis, the SASP could also influence tissue aging. Studies on aging telomere dysfunctional mice have provided direct experimental evidence for an *in vivo* activation of the SASP in response to telomere dysfunction [19]. Interestingly, this *in vivo* SASP provoked alterations in stem cell differentiation (skewing of hematopoiesis towards reduction in lymphopoiesis and enhancement of myelopoiesis) that are also characteristic signs of human aging.

**Figure 1.**
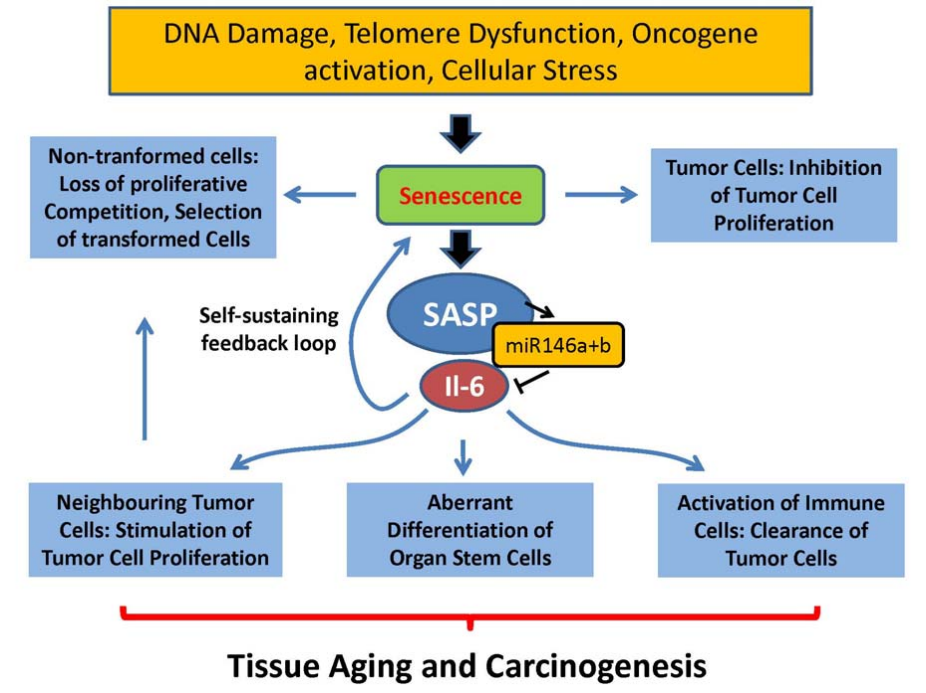
Different cellular stresses can induce senescence including telomere shortening, DNA damage, and oncogene activation. Senescence of tumor cells represents a cell intrinsic tumor suppressor mechanism. In contrast senescence of non-transformed cells in aging organs may lead to loss of proliferative competition and selection of malignant clones. The senescence associated secretory phenotype (SASP) could have different effects on aging and cancer: (i) it could contribute to the induction and maintenance of senescence via a feedback loop, (ii) it could activate immune response leading to improved clearance of senescent tumor cells, (iii) it could stimulate proliferation of neighbouring tumor cells, (iv) it could impair the function of non-transformed tissue stem cells.

In light of the many possible roles o the SASP in aging and carcinogenesis, it appears to be of utmost importance to decipher regulatory pathways controlling the SASP. In a current publication, Bhaumik et al. have identified 2 microRNAs (miR-146a/b) that negatively regulate the secretion of IL-6 and IL-8 - two of the SASP [20].  The authors show that these microRNAs are up-regulated at late stages of senescence, many days after a permanent cell cycle arrest has been established. Interestingly, the inhibitory miRs are most strongly up-regulated in senescence of cell lines that show a strong SASP but not in cell lines characterized by a weak SASP. The authors propose a new concept indicating that miRs 146a and b function in a negative feedback loop preventing an over-activation of the SASP in senescent cells. The authors present some initial data suggesting that activation of this negative feedback loop involves IL-1 receptor, IRAK-1, and NFκB signalling leading to an up-regulation of miRs-146a and b. A direct proof that this proposed feedback loop suppresses over-activation of the SASP remains to be demonstrated in future studies. The authors show that blockage of IL-1-receptor signalling prevents both the up-regulation of miRs-146a and b as well as Il-6 secretion. To confirm their new concept, it would be important to show that a selective blockage of miRs-146a and b results in over-activation of the SASP.

The work by Bhaumik et al. places mir-146a/b as central players to control IL-6 and IL-8 expression within the SASP. MicroRNAs are emerging therapeutic targets because their expression levels can be effectively modulated via the use of antagomirs (see for example [21]). Also, for increasing microRNA expression, microRNAs can be delivered into cells*in vivo* (see for example [22]). Therefore, it will be interesting to functionally test the impact of mir-146 inhibition on tumorigenesis and aging in relevant mouse models. Such studies will be of particular interest, as recent work showed that IL-6 secretion by senescent cells is relevant for initiating and maintaining the senescene response via an autocrine loop [17]. A reduction of miR-146 could increase IL-6 levels in senescent cells, which should stabilize the senescence program and reduce the risk of malignant transformation. Furthermore, it can be speculated that reduction of mir-146 a/b will increase NfκB activation via IRAK1. As NfκB is modulating the expression of various inflammation associated genes, this may also lead to increased clearance of senescent tumor cells by the innate immune system. However, it should be mentioned that Il-6 secreted by senescent cells can also act as a mitogen for surrounding cells, thus potentially increasing the risk of malignant transformation [13,17]. Besides its function in SASP modulation, miR-146 was also reported to target the mRNAs of the BRCA1 and BRCA2 tumor suppressors. In a recent study a G to C polymorphism in miR-146, which leads to an increased processing and release of the mature microRNA, can predict an early onset of breast cancer [23].

Taken together, the study of Bhaumik et al. opens an interesting new research area dealing with the gene regulatory mechanisms that control activation of the SASP. Given the diverse roles of the SASP in modulating tumor progression, immune surveillance of damaged cells, and the stabilization of the senescence arrest itself, it will be of great interest to analyse the influence of SASP regulatory pathways during aging and cancer.
